# The effect of formulation vehicles on the *in vitro *percutaneous permeation of ibuprofen

**DOI:** 10.1186/1471-2210-11-12

**Published:** 2011-12-14

**Authors:** Jessica Stahl, Mareike Wohlert, Manfred Kietzmann

**Affiliations:** 1 Department of Pharmacology, Toxicology and Pharmacy, University of Veterinary Medicine Hannover, Foundation, Buenteweg 17, 30559 Hannover, Germany

## Abstract

**Background:**

The transdermal application of substances represents an elegant approach to overcome side effects related to injections or oral treatment. Due to benefits like a constant plasma level, no pain during application and a simple therapeutic regime, the optimization of formulations for transdermal drug delivery has gained interest in the last decades. Ibuprofen is a non-steroidal anti-inflammatory compound which is nowadays often used transdermally. The objective of this work was to conduct a study on the effect of different 5% ibuprofen containing formulations (Ibutop^® ^cream, Ibutop^® ^gel, and ibuprofen solution in phosphate buffered saline) on the *in vitro*-percutaneous permeation of ibuprofen through skin to emphasise the importance of the formulation on percutaneous permeation and skin reservoir.

**Methods:**

The permeation experiments were conducted in Franz-type diffusion cells according to OECD guideline 428 with 2 mg/cm^2 ^ibuprofen formulation on each skin sample. Ibuprofen was analysed in the receptor fluid and extracted skin samples by UV-VIS high-performance liquid-chromatography at 238 nm. The plot of the cumulative amount of ibuprofen permeated versus time was employed to calculate the apparent permeability coefficient, the maximum flux and the lagtime, all of which were statistically analysed by One-way ANOVA.

**Results:**

Although ibuprofen permeation out of the gel increases rapidly within the first four hours, the cream produced the highest ibuprofen delivery through the skin within 28 hours, followed by the solution and the gel. A significant shorter lagtime was found after gel treatment compared with the cream and the solution. After 28 hours 59% of the applied ibuprofen was found in the receptor fluid of the cream treated samples, 26% in the solution treated samples and 21% in the samples treated with the gel. Fourfold higher ibuprofen reservoirs were found in the solution and gel treated skin samples compared to the cream treated skin samples.

**Conclusion:**

The present study demonstrates the importance of the formulation on transdermal drug delivery of ibuprofen and emphasises the differences of drug storage within the skin due to the formulation. Thus, it is a mistaken assumption that formulations comprising the same drug amount are equivalent regarding skin permeability.

## Background

The skin with a surface area of approximately 2 m^2 ^in human beings represents the largest organ of the body and protects the latter from the intrusion of environmental substances and transepidermal water loss [1]. However, the main skin barrier, the *stratum corneum*, is permeable for a small percentage of compounds with moderate oil-water partition coefficients, small molecular weights and low melting points [2]. The *stratum corneum *is a complex structure comprising hydrophilic "dry" corneocytes embedded in a lipid matrix predominantly consisting of a mixture of ceramides, cholesteryl and free fatty acids [3]. Compounds on the skin surface can pass through the *stratum corneum *by different ways. Beside the intercellular way through the lipid matrix between the corneocytes, hair follicles and sebaceous glands are likely to be employed as diffusion shunt ways [1]. To facilitate transdermal drug delivery for substances without permeation or to increase the amount of substance with poor permeation profiles many studies have been conducted (reviewed in [4]). Beside chemical permeation enhancers such as alcohols or terpenes [4,5] which interact with the *stratum corneum *architecture or lipid composition, physical enhancers like iontophoresis, sonophoresis or microneedles have been produced to overcome the skin barrier [6-9]. Likewise, the formulation in which a compound is applied onto the skin determines the rate of percutaneous permeation [10], as thermodynamic activities e.g. can vary from formulation to formulation. Although *in vivo- *and *in vitro-s*tudies demonstrate the vehicle dependence of the permeation profile of ibuprofen, the variety of ingredients used can barely be used to predict ibuprofen delivery out of these formulations [11,12]. Consequently, comparative studies with effective formulations are necessary to characterise the effectiveness of the marketed products such as creams, gels, sprays, mousses and ointments.

Thus, the object of the present study was to examine the *in vitro *permeation profile of ibuprofen out of two marketed formulations and ibuprofen solution, all of which contained 5% ibuprofen, through skin to show the impact of the vehicle on transdermal drug delivery. Moreover, the ibuprofen content in the skin samples was measured to investigate whether the formulation determines the drug reservoir within the skin.

## Methods

### Chemicals

All reagents used for the present study were of the highest purity available. The salts used for the buffer production were obtained from Merck (Darmstadt, Germany) and methanol was obtained from Applichem (Darmstadt, Germany).

### Membranes

The skin was obtained from udders of Holstein Friesian cows at the slaughterhouse. In brief, skin flaps were removed from udders immediately after slaughter. The flaps were washed with cold water before they were stored at -20°C until use (maximum 3 weeks). Prior to the experiment, the skin flaps were defrosted and an electrical dermatome (Zimmer, Eschbach, Germany) was employed to obtain 500 μm (± 100 μ m) thick split skin samples.

### Ibuprofen formulations

The ibuprofen 5% w/w formulations utilised in the present study were Ibutop^® ^gel (mibe GmbH Arzneimittel, Brehna, Germany) and Ibutop^® ^cream (Dr. Henk Pharma GmbH, Sankt Augustin/Bonn, Germany) as well as an ibuprofen (Sigma-Aldrich, Steinheim, Germany) containing solution (in phosphate buffered saline (PBS, pH 7.4; 1 l contains 0.2 g KCl, 8.0 g NaCl, 0.2 g KH_2_PO_4_, 1.44 g Na_2_HPO_4 _× 2H_2_O and deionised water)). The ingredients of the formulations are presented in Table 1.

**Table 1:** Ingredients of topical formulations containing ibuprofen 5%

	**Ibutop^® ^gel**	**Ibutop^® ^cream**	**Ibuprofen solution**
	
**Active ingredient**	Ibuprofen 5%	Ibuprofen 5%	Ibuprofen 5%
**Excipients**	Dimethyl isosorbide	Sodium methyl hydroxybenzoate	Phosphate buffered saline (pH 7.4)
	2-Propanol	Medium chain triglycerides	
	Poloxamer	Glycerol-monostearate	
	Medium chain triglycerides	Macrogol stearate 1500	
	Lavender oil	Macrogol stearate 5000	
	Orange blossom	Propylenglycol	
	Purified water	Xanthan gum	
		Purified water	

### *In-vitro *permeation experiments

The permeation experiments were conducted in skin samples of four to six animals in duplicate under non-occlusive conditions according to OECD guideline 428 [13]. Prior to the experiment both the skin samples were cut into pieces of 4 cm^2 ^which were incubated in PBS for 30 minutes. They were mounted between the donor and the receptor chamber of Franz-type diffusion cells (with the *stratum corneum *pointing to the donor chamber), all of which were maintained at 32°C by a water bath. The receptor chambers were filled with 12 ml sonicated PBS and stirred continuously with a magnetic bar (500 rpm). Just before administration of the different formulations on the membrane surface a 400 μl sample was withdrawn from the receptor chamber. Additional samples were taken at the following times: 0.5, 1, 2, 3, 4, 6, 22, 24, 26 and 28 hours. After each sample withdrawal the receptor chamber was refilled with 400 μl PBS.

### Ibuprofen measurement in the skin samples

The amount of ibuprofen in the skin samples was determined after completion of the diffusion experiment. Therefore, the skin samples were removed from the diffusion cells and rinsed with cold water. A biopsy-punch with a diameter of 6 mm was employed to obtain a skin biopsy from the diffusion area, which was shaken with 10 μl H_2_SO_4 _(2 N) and 800 μl methanol for 30 minutes. After 24 hours incubation at 4°C the supernatant was collected and 190 μl McIlvaine citrate buffer (pH 2.2; 1 l contains 20.8 g citric acid anhydrous, 0.4 g Na_2_HPO_4 _× 2H_2_O and deionised water) were added. The recovery was approximately 85-90%.

### Analysis

Ibuprofen in both the receptor medium samples and the skin samples was analysed by a validated UV-VIS high-performance liquid-chromatography methodology using a Merck LiChroCART 125-4 Lichrospher 100 RP18e (5 mm) column (Darmstadt, Germany) and a model 168 UV-VIS detector from Beckman (Fullerton, CA, USA) at 238 nm [14]. The mobile phase which was a 80:20 (v/v) mixture of methanol and McIlvaine citrate buffer (pH 2.2) was pumped by a 116 pump from Beckman (1.5 ml/min). The content of ibuprofen was determined using the external standard method. The limit of quantification was 200 ng/ml and the limit of detection was 100 ng/ml. The recovery results in the receptor chamber are expressed as percentage recovery of the applied amount of ibuprofen. The amount of ibuprofen found in the skin samples is expressed as "skin absorption" in μg/cm^2^.

### Data analysis

The plot of the cumulated amount of ibuprofen versus time was employed for the calculation of the apparent permeability coefficients (P_app_) according to Niedorf et al. 2008 [15] as well as for the determination of the maximum flux J_max _and the lagtime. Values from replicated experiments (2 replicates per formulation) in skin from the same individual were used as average. Differences in the permeation parameters (P_app_, J_max_, lagtime, and recovery) were evaluated using One-way ANOVA (analysis of variance) followed by Tukey's multiple comparison test (GraphPad Prism 4.01 (GraphPad Software Inc., San Diego, USA)) with a significance level of p < 0.05.

## Results

**Figure 1:**
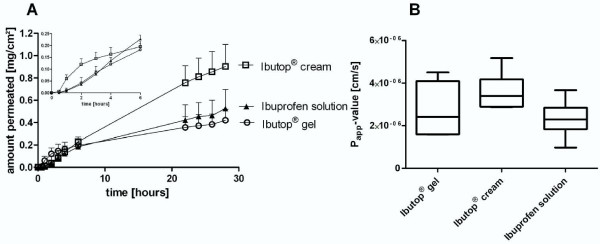
**Skin permeation of ibuprofen**. A: Ibuprofen permeation of bovine udder skin samples *in vitro *following application of topical formulations containing ibuprofen 5% (mean + standard deviation) and B: the obtained apparent permeability coefficients (boxplots with minimum and maximum; n = 5-6).

After administration of three different ibuprofen containing formulations on bovine udder skin substantial differences in the permeation profiles were obtained (Figure 1A). The highest ibuprofen permeation was found after application of the cream formulation followed by a moderate ibuprofen permeation out of the solution and the lowest ibuprofen delivery out of the gel, despite a sharp increase of the ibuprofen permeation within the first two hours after gel administration. Nevertheless, the P_app_-values obtained from the linear part of the plot were not significantly different between the groups (Figure 1B and Table 2). After 28 hours significant higher ibuprofen contents were found to have been passed through the skin after topical administration of the cream (Table 2) by comparison to the gel and the solution. However, the highest ibuprofen reservoir in the skin samples was found in the samples treated with the solution, closely followed by the gel, while fourfold lower amounts of ibuprofen were determined in the cream treated skin samples (Table 2). Due to a high standard deviation, these differences were not statistically significant. Significant shorter lagtimes were found after gel application in comparison to the administration of the cream or the solution (Table 2).

**Table 2:** Skin permeation parameters

**Formulation**	**Recovery (%)**	**J_max _(μ g/cm^2^/h)**	**Lagtime (h)**	**Skin absorption (μ g/cm^2^)**
	
**Ibutop^® ^gel**	21 ± 5^a^	50 ± 23	0.0 ± 0.2^ac^	239 ± 159
**Ibutop^® ^cream**	59 ± 8	65 ± 15	1.3 ± 0.6	57 ± 25
**Ibuprofen solution**	26 ± 8^b^	42 ± 16	1.3 ± 0.8	264 ± 192

Absorption parameters of bovine udder skin (recovery in the receptor chamber, J_max_, lagtime, and skin absorption) following application of different topical formulations containing ibuprofen 5%; n = 5-6, mean and standard deviation; p < 0.05 (One-way ANOVA followed by Tukey's multiple comparison test; a: gel vs. cream, b: solution vs. cream, c: gel vs. solution)

## Discussion

The present study was conducted to investigate the influence of the galenic formulation on the percutaneous permeation of ibuprofen and its storage within the skin. Although the examined formulations comprised the same amount of ibuprofen (5% w/w), considerable differences in the permeation profiles were determined comparable with Hadgraft et al. (2003) [11]. The cream formulation was the most efficient vehicle for ibuprofen permeation through the skin as it reached twofold to threefold higher amounts of ibuprofen in the receptor chamber within 28 hours. The permeation of the ibuprofen solution ranged between the permeation of the cream and of the gel which offered a lower ibuprofen delivery through the skin. Despite a sharp initial increase in the permeation of ibuprofen out of the latter, only a small percentage of ibuprofen was delivered into the receptor fluid after application of the gel formulation over time. It has to be taken into consideration that ibuprofen represents a non-steroidal anti-inflammatory drug which is predominantly taken due to its rapid pain relief and anti-inflammatory action. Thus, a high percentage of permeated ibuprofen results in the best clinical benefits. It is likely, that the application of the gel leads to a fast and effective ibuprofen concentration in the organism, whereas the data of the present *in vitro *study do not inform about effective plasma levels after topical administration *in vivo*. It has to be discussed whether the sharp incline of the ibuprofen permeation out of the gel is prejudicial to the patient compared to a steady ibuprofen permeation in the first hours after administration. Since high amounts of ibuprofen were found to be in the skin after 28 hours gel treatment, it can be supposed that, on condition that the comparatively low permeation is sufficient to produce efficacious blood levels, the gel offers advantages concerning a long-term treatment. Thus, the skin supplies the body continuously with ibuprofen without the need of a follow-up treatment, even after removal of the remained formulation on the skin surface.

The fact that different formulations comprising ibuprofen as active compound impact the amount of transdermal drug delivery confirms findings of Hadgraft et al. 2003 [11] and is likely to be explained by partition and diffusion phenomena. Therefore, 5% ibuprofen containing formulation applied onto the skin can result in unpredictable ibuprofen fluxes, which have to be compared to effective therapeutics. Furthermore, the drug reservoir within the skin is considerably determined by the galenic formulation. Surprisingly, the topical application of the self-made ibuprofen solution as control resulted in a permeation rate in the middle of the marketed products, despite the lacking of supplementation of permeation enhancers like 2-propanol or medium chain triglycerides, all of which interact with the *stratum corneum*.

## Conclusion

The present investigation demonstrates the importance of vehicle formulation on transdermal drug delivery of ibuprofen and highlights the differences of drug reservoirs within the skin due to different galenics.

## Competing interests

The authors declare that they have no competing interests.

## Authors' contributions

JS designed the study, conducted the extraction experiments, contributed to the analysis, interpreted results and drafted the manuscript. MW participated in the diffusion experiments. MK participated in the study design development. All authors have read and approved the final manuscript.
